# Draft Genome Sequence of *Actinomadura madurae* LIID-AJ290, Isolated from a Human Mycetoma Case

**DOI:** 10.1128/genomeA.00201-14

**Published:** 2014-03-27

**Authors:** Lucio Vera-Cabrera, Rocio Ortiz-Lopez, Ramiro Elizondo-González, Mayra Paola Campos-Rivera, Anabel Gallardo-Rocha, Carmen Amelia Molina-Torres, Jorge Ocampo-Candiani

**Affiliations:** a Laboratorio Interdisciplinario de Investigación Dermatológica, Servicio de Dermatología, Hospital Universitario, UANL, Monterrey, México; b Universidad Autónoma de Nuevo León, Departmento de Bioquímica y Medicina Molecular, Monterrey, México; c Universidad Autónoma de Nuevo León, Centro de Investigación y Desarrollo en Ciencias de la Salud, Monterrey, México

## Abstract

Here we present the draft genome sequence of a member of the *Thermomonosporaceae*, *Actinomadura madurae* LIID-AJ290, isolated from a human case of mycetoma. The assembly contains 10,308,866 bp. This is to our knowledge the first reported genome of a human-pathogenic *Actinomadura* species.

## GENOME ANNOUNCEMENT

Mycetoma is a subcutaneous infection produced by fungi and aerobic actinomycetes. It is characterized by tumefaction of the affected region, production of abscesses and fistulae, and the presence of microcolonies of the etiologic agent. Although worldwide 50% of mycetoma is produced by eumycetes and 50% by actinomycetes (1), in Mexico actinomycetoma is more commonly reported, accounting for about 98% of the cases. *Nocardia brasiliensis* is found in 86% of the infections, followed by *Actinomadura madurae* and *Nocardia* spp., which produce the rest of the cases (2). The genome sequence of *N. brasiliensis* has been reported (3, 8). Herein, we report the first draft of the genomic sequence of *A. madurae.*

*A. madurae* LIID-AJ290 was isolated in 2011 from a mycetoma case in the foot. The isolate has <20 subcultures. The bacteria used for genome sequencing were isolated from a purified stock kept at −70°C. The bacterial culture was expanded on brain heart infusion at 37°C with shaking at 110 rpm for 72 h, and the bacterial mass was subjected to DNA extraction using the cetyltrimethylammonium bromide (CTAB) method. The bacterial identity was determined using a hydrolysis pattern (4) as well as nucleotide sequencing analysis of a fragment of the 16S RNA gene using primers NOC3 and NOC4 (5). The genome sequence was determined using the platforms Roche/454 GS Junior (428,248 reads) and Illumina/MiSeq (1,243,587 reads). Both sets of reads were combined and assembled using Newbler 2.7 software (Roche Diagnostics, Branford, CT).

The genome annotation was carried out using the NCBI Prokaryotic Genomes Automatic Annotation Pipeline (PGAAP). According to the NCBI, the PGAAP combines hidden Markov model (HMM)-based gene prediction methods with a sequence similarity-based approach which combines comparison of the predicted gene products with the nonredundant protein database Entrez Protein Clusters, the Conserved Domain database, and the Clusters of Orthologous Groups (COGs) database (6, 7).

The unclosed draft genome of *A. madurae* LIDD-AJ290 consists of 149 contigs and is a total of 10.30 Mbp long, with a G+C content of 72.04%. According to the GenBank annotation system, the draft genome contains 9,643 genes, 10 rRNAs, 80 tRNAs, and 1 CRISPR array.

### Nucleotide sequence accession numbers.

This whole-genome shotgun project has been deposited at DDBJ/EMBL/GenBank under the accession number AWOO00000000. The version described in this paper is version AWOO02000000.

## References

[1] Mariat F Destombes P Segretain G 1977 The mycetomas: clinical features, pathology, etiology and epidemiology. Contrib. Microbiol. Immunol. 4:1–39 342189

[2] Lopez-Martinez R Méndez Tovar LJ Lavalle P Welsh O Saúl A Macotela-Ruiz E 1992 Epidemiology of mycetoma in Mexico: study of 2105 cases. Gac. Med. Mex. 128:477–481 (In Spanish.) 1308000

[3] Vera-Cabrera L Ortiz-Lopez R Elizondo-Gonzalez R Perez-Maya AA Ocampo-Candiani J 2012 Complete genome sequence of *Nocardia brasiliensis* HUJEG-1. J. Bacteriol. 194:2761–2762. 10.1128/JB.00210-12 22535940 PMC3347167

[4] Koppenstedt RM Goodfellow M 2006 The family *Thermomonosporaceae*: *Actinocorallina*, *Actinomadura*, *Spirillospora* and *Thermomonospora*. *In* Dworkin F Falkow S Schleifer KH Stackebrandt E (ed), The prokaryotes: a handbook on the biology of bacteria, vol 3 Springer-Verlag, New York, NY.

[5] Vera-Cabrera L Johnson WM Welsh O Resendiz-Uresti FL Salinas-Carmona MC 1999 Distribution of a *Nocardia brasiliensis* catalase gene fragment in members of the genera *Nocardia*, *Gordona*, and *Rhodococcus*. J. Clin. Microbiol. 37:1971–1976 10325357 10.1128/jcm.37.6.1971-1976.1999 PMC84999

[6] Borodovsky M McIninch J 1993 GeneMark: parallel gene recognition for both DNA strands. Comput. Chem. 17:123–133. 10.1016/0097-8485(93)85004-V

[7] Delcher AL Harmon D Kasif S White O Salzberg SL 1999 Improved microbial gene identification with GLIMMER. Nucleic Acids Res. 27:4636–4641. 10.1093/nar/27.23.4636 10556321 PMC148753

[8] Vera-Cabrera L Ortiz-Lopez R Elizondo-Gonzalez R Ocampo-Candiani J 2013 Complete genome sequence analysis of *Nocardia brasiliensis* HUJEG-1 reveals a saprobic lifestyle and the genes needed for human pathogenesis. PLoS One 8:e65425. 10.1371/journal.pone.0065425 23755230 PMC3670865

